# Ultra-Thin and Broadband P-Band Metamaterial Absorber Based on Carbonyl Iron Powder Composites

**DOI:** 10.3390/ma17051157

**Published:** 2024-03-01

**Authors:** Mengyu Zhou, Yubin Chen, Yuguang He, Cheng Yang

**Affiliations:** 1 AECC Beijing Institute of Aeronautical Materials, Beijing 100095, China; zhoumengyu0704@163.com (M.Z.); himuman@163.com (Y.H.); 2 Beijing Institute of Graphene Technology, Beijing 100094, China

**Keywords:** metamaterial absorber, P-band, electromagnetic parameters, broadband absorption

## Abstract

The field of P-band (0.3–1 GHz) absorption has witnessed rapid development in metamaterial absorbers due to their exceptional designability and the absence of restrictions imposed by the one-fourth wavelength rule. In this study, we combined carbonyl iron powder (CIP) composites with a periodic structure composed of metal capacitive patterns and employed a genetic algorithm (GA) to optimize the electromagnetic parameters of the CIP substrate. By selecting the appropriate shape and material for the units of pattern based on transmission line theory, as well as regulating relevant structural parameters, we successfully designed an ultra-thin broadband metamaterial absorber for the P-band. Experimental results demonstrate that within the range of 0.3–0.85 GHz, the reflection loss of our absorber remains below −5 dB, with a maximum value of −9.54 dB occurring at 0.45 GHz. Remarkably, this absorber possesses a thickness equivalent to only 1/293 of its working wavelength. Then, we conducted analyses on electric field distribution, magnetic field distribution, and energy loss density. Our findings suggest that high-performance absorption in metamaterials can be attributed to λ/4 resonant or coupling effects between structural units or diffraction phenomena. This absorber offers several advantages, including broad low-frequency absorption capability, ultra-thin profile, and convenient fabrication process, thus providing valuable theoretical insights for designing metamaterial structures.

## 1. Introduction

With the rapid advancement of detection technology and precision guidance systems, the significance of stealth capabilities in aviation weaponry has become increasingly prominent [1]. High-performance absorbing materials play a crucial role in achieving stealth technology [2]. Although significant research progress has been made on wide-band absorbing materials, extending even to the L-band frequency range (1–2 GHz) nowadays [3,4], the longer wavelengths present a formidable challenge for electromagnetic parameter optimization and thickness control of absorbing materials when deploying new long-range early warning radars operating at lower frequencies, such as P-band [5,6].

According to the transmission line theory, an ideal P-band absorbing material requires an extremely high real and imaginary part of permeability, a high real part of the dielectric constant, and a low imaginary part of the dielectric constant [7]. To address the issue that common materials cannot meet the requirements of P-band absorbing properties, some researchers have explored enhancing the magnetic properties through composite, nanocrystalline/amorphous, and particle spatial morphology control. V.G. Andreev et al. discovered that incorporating 51.0 mol% Fe_2_O_3_ can elevate the magnetic permeability of Ni-Zn ferrite to 1200, enabling ultra-high electromagnetic parameters for superior P-band absorption performance [8]. Igor Isaev et al. synthesized [9] Li_0.33_Fe_2.29_Zn_0.21_Mn_0.17_O_4_ spinel ferrite using ceramic technology, which can effectively absorb electromagnetic radiation in the frequency range of 0.05–7.0 GHz. However, due to increasingly intricate synthesis processes and stringent preparation condition controls, the application scale-up potential for these magnetic materials used in the P-band remains limited [10,11].

Benefiting from their strong designability, easy adjustability, and freedom from the limitations imposed by one-fourth wavelength rules, metamaterial absorbers have experienced rapid development in the field of low-frequency absorption [12,13]. Active metamaterial absorbers demonstrate enhanced electromagnetic absorption through the integration of electrical components [14]. Yifeng Fan et al. designed active-loaded metamaterials employing NFC (non-Foster circuit) based on RTD (resonant tunneling diodes), enabling broadband and wide-angle absorption in the P-band [15]. Weiqingzuo et al. introduced lumped resistors to metal-bending structures to fabricate ultra-wideband metamaterial absorbers with an absorption rate exceeding 90% within the 0.8–2.7 GHz band and a relative bandwidth surpassing 108% [16]. However, it is worth noting that the introduction of electronic components may give rise to chaotic electronic fields and diminished intensity, thereby imposing limitations on their application as absorbers in aviation and aircraft.

Despite the absence of electrical components, passive metamaterials can effectively fulfill the requirements for low-frequency absorption through well-designed surface structures [17]. However, a challenge remains in terms of achieving narrow absorption frequency bands [18,19]. Typically, magnetic components are combined with these materials to address this issue and capitalize on the advantages offered by different materials or structural elements. For instance, Yang Jingxian et al. [20] developed square ring metamaterials integrated with magnetic materials to create a composite absorber measuring 4.5 mm in thickness. This absorber exhibited reflectivity lower than −8 dB within the frequency range of 400–600 MHz and as low as −10 dB at 468–495 MHz. Longhui He et al. achieved an effective absorption bandwidth from 210 MHz to 1000 MHz by incorporating cross-shaped metamaterials into NiZn ferrite [21], resulting in overlapping effects from three absorption peaks originating from multi-layered structures. Jiayan Song et al. [22] designed a magnetic fractal metamaterial composite absorber that achieved over 90% absorption in the P-band while also displaying excellent adaptability to various incident angles. The combination of metamaterials and magnetic materials has garnered significant attention and development. However, current research often solely presents metamaterial structures without delving into design principles and selection criteria; further research and improvement are necessary regarding theory, materials, and structural design, among other aspects. Moreover, the metamaterial structures they design often exhibit intricacy and substantial thickness (>10 mm) or remain in the conceptual stage without empirical validation.

In this paper, firstly, the electromagnetic parameters of the basal layer were optimized using a GA based on measured values of absorption performance in order to address the challenges associated with accurately testing the electromagnetic parameters of magnetic materials in the P-band. Then, dielectric theory and material characteristic analysis were integrated to facilitate meticulous design guided by theoretical principles. The absorption characteristics of metamaterials were simulated, the optimal material selection and structural parameters were explored, and a sample was produced for experimental verification. This study is instrumental in designing ultra-thin P-band absorbers with a simplified preparation process and enhanced practicality, thereby presenting significant application prospects in the domain of military weaponry and equipment.

This paper is divided into several sections: First, the selection of magnetic materials (CIP) and correction of material parameters for the basal layer. Secondly, the choice of material types and determination of structural types for pattern layers. Thirdly, the design and optimization for metamaterials and exploration of the influence of relevant structural parameters on absorption performance. Finally, an examination of the absorption mechanism based on the surface electric field, magnetic field, and energy loss distribution. 

## 2. Materials and Methods

### 2.1. Sample Preparation

The CIP composites are a mixture of FeSiCr alloy powder and carbonyl iron powder; they were dispersed and pressed with a mixture of polyurethane and epoxy resin to produce samples of varying thicknesses (1 mm, 2 mm, 3 mm, and 4 mm), which were then cut into films measuring 1 m × 1 m as basal layer. Pattern layers were made using tape lamination and mechanical cutting techniques. Ultimately, the pattern layer was integrated with the magnetic film to form a composite absorber.

### 2.2. Reflectance Testing

The P-band reflectivity of the absorber sample was assessed using the microwave anechoic chamber method. The microwave anechoic chamber, consisting of a combination of specialized absorbers (SA) and metal shielding, creates artificially open conditions resembling ‘free space’.

### 2.3. Simulation and Methods

The unit structure of metamaterial absorbers was designed using the 3D electromagnetic simulation software CST Microwave Studio [23], and a frequency domain solver based on the finite element method was employed for simulation. To facilitate subsequent periodic structural analysis, the XY direction was set as the unit cell boundary condition, while the outer Z direction was set as an open (added space) boundary condition. The absorption rate of the material is represented by *A*, where $A=1-{\left|S_{11}\right|}^{2}-{\left|S_{12}\right|}^{2}$. S_11_ and S_21_ denote the magnitude of return loss and insertion loss, respectively. Since a metal plate is added to the bottom layer of the absorber, preventing electromagnetic waves from passing through it, the absorption rate can be expressed as $A=1-{\left|S_{11}\right|}^{2}$. In the P-band, when $S_{11}$ < −5 dB, the material can be considered to exhibit a high absorption rate. 

### 2.4. Genetic Algorithm

The electromagnetic parameters were optimized using GA [24], a stochastic global search optimization method that employs random selection, crossover, and mutation operations to generate improved solutions with an increased likelihood of selection. Through iterative processes, the optimal solution for the given problem can be found. 

## 3. Results and Discussions

### 3.1. Optimization of Electromagnetic Parameters of Magnetic Materials for Basal Layer

**Figure 1:**
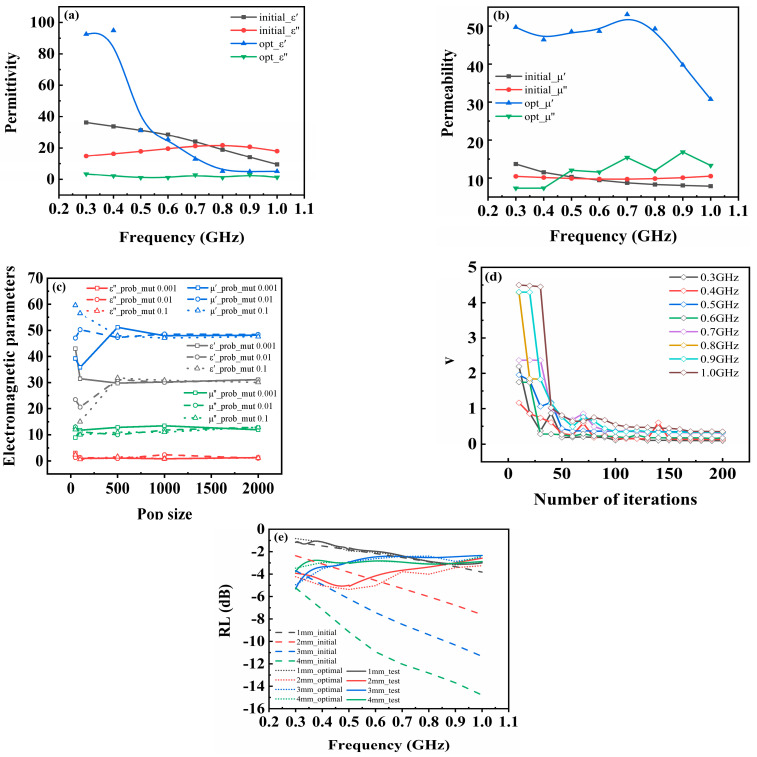
The measured and optimized values of electromagnetic parameters for CIP composites (**a**) permittivity and (**b**) permeability. (**c**) The electromagnetic parameters under different mutation probabilities and population sizes. (**d**) The variation in the objective function value with the number of iterations. (**e**) Experimental values, initial calculated values, and optimized values of reflectivity.

Accurate determination of electromagnetic parameters is crucial and fundamental for achieving precise design and optimization of the absorber. However, direct measurement of electromagnetic parameters in the P-band often suffers from inaccuracies. For instance, the reflectance value calculated from the measured electromagnetic parameters exhibits a significant disparity compared to the experimental value in terms of trend and magnitude, as depicted in Figure 1d. This study is based on the relationship between reflectivity and electromagnetic parameters within the theoretical model [25], which can be found in Supplementary Materials. A GA was employed to iteratively optimize four key parameters (ε’, ε”, μ’, μ”) until the calculated reflectance value matches the experimental value.

Four arguments need to be discussed in a GA: population size, mutation probability, number of iterations, and objective function value v. First, we defined $v=v_{1}+v_{2}+v_{3}+v_{4}$, where $v_{1}$, $v_{2}$, $v_{3}$, and $v_{4}$ represents the difference between calculated and measured reflectance values for magnetic materials with thicknesses ranging from 1 mm to 4 mm, respectively. Moreover, $v_{i}=10^{R/10}$, *R* denotes reflectivity; through mathematical processing, the negative *R* value was converted into numbers ranging from 0 to 1 to facilitate comparison and enhance convergence of the solution. Taking the case at 0.4 GHz as an illustrative example, Figure 1b illustrates the electromagnetic parameters obtained by solving for different population sizes and mutation probabilities, and it is evident that for population sizes below 500, the solutions obtained from different mutation probabilities exhibit significant variation. However, as the population size surpasses 1000, the solution set expands, thereby augmenting the probability of discovering global minima. The resulting solution values are stable and yield superior optimization outcomes. The variation in the objective function value with the number of iterations at different frequencies is depicted in Figure 1c. When exceeding 100 iterations, a convergence trend can be observed where the objective function value stabilizes below 0.5. Converted to reflectivity, the maximum difference observed in a single flat panel is merely 0.75 dB.

Based on the above discourse on arguments, the upper and lower limits (0, 100) of the unknown solution were selected, with a population size ranging from 1000 to 2000, 100 iterations, and a mutation probability of 0.1. The optimized electromagnetic parameters are depicted as the solid line in Figure 1a, demonstrating consistent calculated reflectance values (shown as dot lines in Figure 1d) with the experimental data. Furthermore, upon comparing the optimized electromagnetic parameters with the reflectivity, it can be inferred that higher ε’, μ’ within the frequency range of 0.3–0.6 GHz contribute to specific absorption performance observed with a thickness of 2 mm.

### 3.2. Selection of Material Type and Determination of Structural Type for Pattern Layer

The filtering frequency of the pattern layer is related to the electromagnetic parameters, position, and thickness of the basal layer. When magnetic materials are unilaterally loaded onto the pattern layer, its resonant frequency approximately corresponds to $f=\frac{f_{0}}{\sqrt{\frac{1+e}{2}}}$; $f_{0}$ represents the resonant frequency of the pattern layer, and e denotes the permeability (μ) or permittivity (ε) of the basal layer. At a frequency of 0.3 GHz, with approximate CIP composites being 92.63, there is a rightward shift in the resonant frequency of the pattern layer to 2.05 GHz.

**Figure 2:**
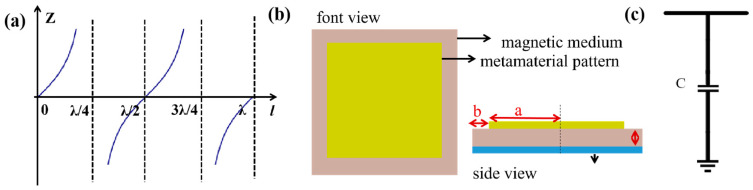
(**a**) Curve of input impedance *Z* with distance *l*. (**b**) Schematic diagram of metamaterial. (**c**) Schematic diagram of equivalent circuit.

After determining the resonant frequency of the pattern layer, transmission line theory [26] can be applied to ascertain whether the pattern layer should exhibit capacitance or inductance. The transmission line theory states that when the terminal load is short-circuited, the input impedance Z at the short circuit distance from the terminal is $Z=jZ_{0}\tan\beta l$, *β* is the phase shift, and $Z_{0}=377\;\Omega$. Figure 2a depicts the variation of input impedance *Z* with distance *l*, showing that the capacitance and inductance of the input impedance cancel each other out at l=λ/4. However, due to the long wavelength of the P-band, fabricating a thickness of λ/4 (75 mm) for the absorber becomes impractical in engineering applications. When the thickness is less than λ/4, it leads to an overall increase in the resistance value of the absorber, necessitating the utilization of a pattern layer to introduce capacitive impedance and alleviate constraints on thickness [27].

The simplest capacitive pattern layer is a square resonant element, approximated as a capacitance C [28,29] in the equivalent circuit (Figure 2c). The schematic diagram of metamaterial is shown in Figure 2b, where the top layer consists of a square pattern with a side length 2a and a gap width 2b, while the lower layer comprises a magnetic substrate, and the bottom layer serves as a metal bottom plate reflector. The relationship between the size of structural units and the occurrence of standing wave resonance is expressed by the following equation: $2a\approx (2j-1)\frac{c}{2n(f_{1})f_{1}}$ where j represents the modulus of standing wave resonance, *n* denotes the refractive index of the basal layer, and $f_{1}$ is resonance frequency, GHz. In the case of resonance mode 1, it can be simplified as $2a\approx \frac{\lambda }{2n\sqrt{e}}$. As the size of the pattern increases, there is a corresponding decrease in resonant frequency. When incorporating a substrate material, the unit size of the pattern is typically close to λ/11, which approximately equals 3 cm.

**Figure 3:**
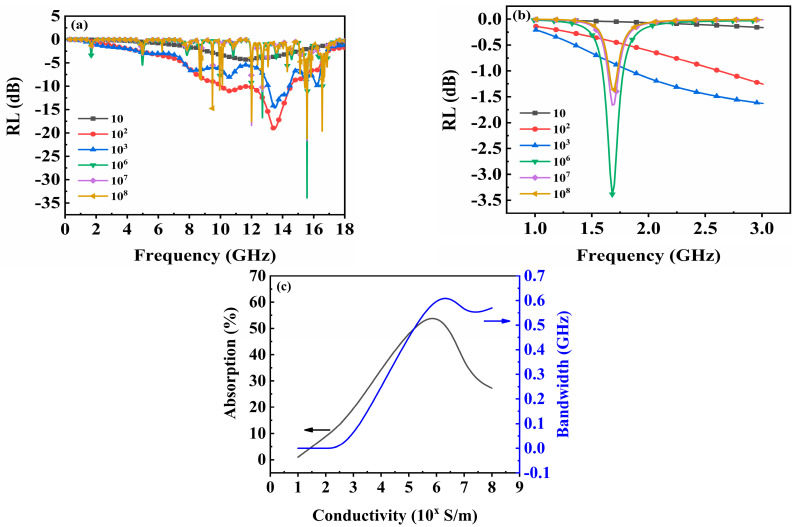
(**a**) Reflectivity at various levels of conductivity within the frequency range of 1–18 GHz. (**b**) Reflectivity at different conductivity levels within the frequency range of 1–3 GHz. (**c**) Curve of absorption rate and bandwidth as a function of conductivity.

The material of the pattern layer also has a significant impact on the absorption performance. In CST, a square pattern with dimensions of 50 × 50 × 0.05 mm was constructed to explore the effect of different conductivities [30] (ranging from 10 to 10^8^ S/m) of the pattern layer on the absorption performance. The lower substrate consists of FR4 with a thickness of 3 mm, a relative dielectric constant of 3.5, and a loss angle tangent of 0.025. Figure 3a illustrates the reflectivity at various conductivity levels within the frequency range of 1–18 GHz. Weak absorption performance is observed at a conductivity level of 10 S/m. Broadband absorption occurs in the range of 8–16 GHz for the conductivities of patterns ranging from 10^2^ to 10^3^ S/m. On the other hand, narrowband and strong absorption occur in different frequency bands for conductivities between 10^6^ and 10^8^ S/m. Particularly when focusing on frequencies between 1 and 3 GHz (Figure 3b), only materials with a conductivity exceeding 10^6^ S/m exhibit absorption resonance peaks. Figure 3c presents the results for both absorption rate and bandwidth at different conductivity levels in the pattern layer. As conductivity increases, both parameters initially increase until reaching their peak values at a conductivity level of 10^6^ S/m; thereafter, they either decrease or remain stable.

This study focuses on the absorption of low-frequency waves, thus necessitating the selection of materials with high conductivity. However, it is challenging to obtain a material with the optimal calculated conductivity of 10^6^ S/m in experiments. Therefore, copper, commonly used and easily obtainable with a conductivity of 5.8 × 10^7^ S/m, is chosen as the material for the pattern layer.

### 3.3. The Influence of Metamaterial Structural Parameters on Absorption Performance

**Figure 4:**
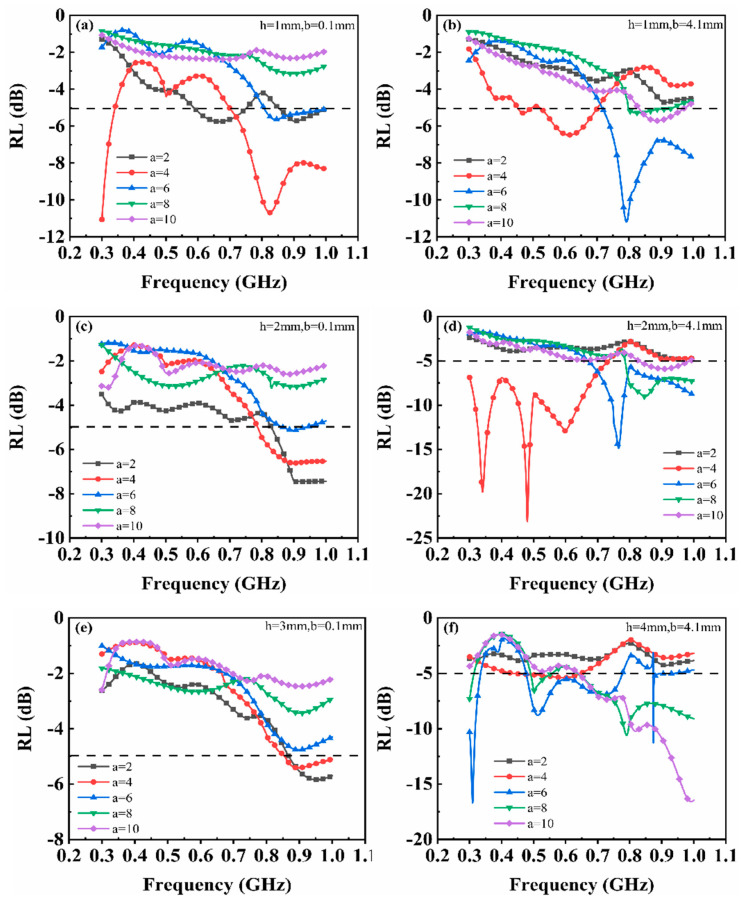
The reflection loss curves for different ‘a’ values (ranging from 2 to 10 mm) in CST: (**a**) h = 1 mm, b = 0.1 mm; (**b**) h = 1 mm, b = 5 mm; (**c**) h = 2 mm, b = 0.1 mm; (**d**) h = 2 mm, b = 5 mm; (**e**) h = 3 mm, b = 0.1 mm; and (**f**) h = 4 mm, b = 5 mm.

The electromagnetic parameters of the material were inputted into CST to calculate the reflection loss (RL) curves. Subsequently, the influence of metamaterial structural parameters (a, b, and h) on absorption performance was explored, and optimal parameters for P-band absorption were identified. The half-length ‘a’ of units ranged from 1 mm to 15 mm with an interval of 1 mm, while the half-gap ‘b’ ranges from 0.1 mm to 5 mm with an interval of 0.5 mm. The magnetic substrate thickness ‘h’ was set at values of 1 mm, 2 mm, 3 mm, and 4 mm. In Figure 4, the dashed line represents the reference line for P-band absorption; structures exhibiting calculated curves below this line can be considered as possessing high-performance absorption.

In Figure 4a, with h = 1 mm and b = 0.1 mm, narrow absorption peaks are observed around 0.65 GHz and 0.9 GHz when a = 2 mm. Resonance peaks appear at 0.3 GHz and 0.8 GHz when a = 4 mm. When the gap ‘b’ is increased to 5 mm, as depicted in Figure 4b, no resonance absorption effect is observed for a = 2 mm. However, significant absorption resonance peaks are generated at 0.6 GHz and 0.8 GHz for a = 4 mm or 6 mm, respectively. For h = 1 mm (shown in Figure 4a,b), no resonance is observed in the P-band for either a = 8 mm or 10 mm. When h = 2 mm, a marginal absorption effect is observed at 0.4 GHz for the single-layer magnetic substrate. Upon introducing square units in metamaterials with dimensions of b = 5 mm and a = 4 mm, resonance occurs at frequencies of 0.3, 0.5, and 0.65 GHz. These three absorption peaks are coupled, resulting in a reflection loss below −5 dB within the frequency range of 0.3–0.7 GHz. The effective absorption bandwidth measures 0.4 GHz, with a minimum reflection loss of −24.04 dB observed at a frequency of 0.48 GHz. When b = 0.1 mm, only when a = 2 mm does an absorption effect occur within the frequency range of 0.8–1 GHz; however, combining the pattern layer with other structural parameters yields negative effects. For h = 3 mm and b = 0.1 mm, similar absorption characteristics are observed for h = 2 mm (Figure 4e). Finally, when h = 4 mm, absorbers with dimensions of a = 2 or 4 mm exhibit no significant absorption effect, whereas for patterns with dimensions of a = 6, 8, or 10 mm, the metamaterials combined with the substrate layer produce narrow absorption peaks within specific frequency bands.

The influence of a single parameter ‘a’ on the overall absorption performance is not monotonic but rather intricately intertwined with the other two parameters. For instance, when h = 1 mm and b = 0.1 mm, a smaller value of ‘a’ will generate resonance at a certain frequency point. Conversely, when h = 4 mm and b = 5 mm, a larger value of ‘a’ results in resonance occurring at certain frequency points.

Based on the results shown in Figure 4, the optimal results in terms of effective absorption bandwidth and maximum reflection loss for the metamaterial are observed when a = 4 mm, b = 5 mm, and h = 2 mm among the aforementioned schemes. To investigate the impact of pattern layer gap on the absorption performance of metamaterials, we fixed a = 4 mm and h = 2 mm while varying the value of ‘b’ from 0.1 to 5 mm.

**Figure 5:**
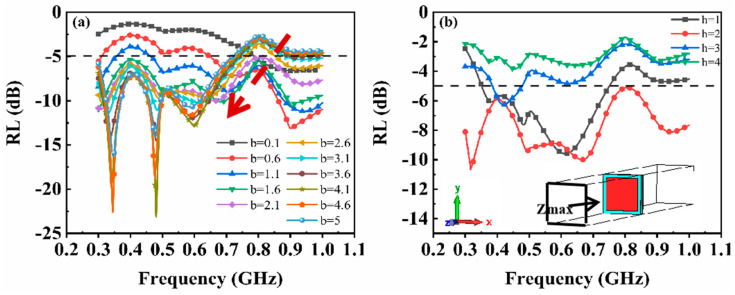
The reflection loss curve calculated in CST: (**a**) h = 2 mm, a = 4 mm, a = 0.1–5 mm; (**b**) h = 1–4 mm, a = 4 mm, b = 1.5 mm.

In Figure 5a, it can be seen that within the frequency range of 0.3–0.6 GHz, an increase in gap size leads to a gradual decrease in the RL value of the absorber, accompanied by distinct and sharp resonance peaks at 0.34 GHz and 0.49 GHz. The absorption peak at 0.34 GHz shifts towards higher frequencies with increasing gap size, whereas the absorption peak at 0.49 GHz shifts towards lower frequencies as the gap increases. Additionally, an absorption peak is observed in the frequency range of 0.6–0.8 GHz, which also exhibits a downward shift with increasing frequency values. Simultaneously, there is a gradual increase in maximum reflection loss for the absorber within the range of 0.3–0.8 GHz as the gap increases. On the other hand, within the frequency range of 0.8–1 GHz, an upward trend can be observed in terms of RL value for larger gaps.

In transmission line theory, the capacitance of a square resonant unit is $C=\varepsilon_{0}\varepsilon_{r}\frac{2a}{\pi }In(\frac{1}{\sin\frac{\pi b}{2a}})$; as the unit size increases, the gap between square rings decreases, resulting in a decrease in equivalent capacitance and a shift of the absorption frequency band towards lower frequencies. Additionally, when a square resonant unit is superimposed on a magnetic material, mutual resonance occurs, resulting in multiple absorption peaks where both parameters (a and b) are coupled together. Therefore, the effect of gaps on the shift of absorption peaks and the maximum absorption loss exhibits relatively intricate behavior. Specifically, when b = 0.1–1.1 mm, the RL value exceeds −5 dB within the frequency of 0.3–0.8 GHz but falls below −5 dB within 0.8–1.0 GHz; when b = 2.6–5 mm, an opposite absorption effect is observed with the RL value less than −5 dB in 0.3–0.8 GHz and no absorption effect in 0.8–1.0 GHz. Only at b = 1.5 or 2.1 mm does the RL value remain below −5 dB across the entire frequency range of 0.3–1.0 GHz.

Furthermore, we fixed the dimensions of a = 4 mm and b = 1.5 mm while varying the thickness h of the magnetic substrate from 1 to 4 mm in order to investigate its impact on the absorption performance of the metamaterial absorbers (Figure 5b). At a thickness of h = 1 mm, the metamaterial exhibits a maximum reflection loss of −9.4 dB at a frequency of 0.62 GHz, with RL values ranging from 0.36 to 0.75 GHz being less than −5 dB. The effective absorption bandwidth is measured as 0.39 GHz. When h increases to 2 mm, three absorption peaks are observed at frequencies of 0.32 GHz, 0.49 GHz, and 0.68 GHz, respectively, with a maximum reflection loss value reaching up to −11.3 dB. Furthermore, an extended effective absorption bandwidth covering the entire P-band is achieved under these conditions.

In summary, it can be concluded that optimal comprehensive absorption performance is obtained when employing dimensions of h = 2 mm, a = 4 mm, and b = 1.5 mm for the metamaterial absorber design scheme. However, when considering thicker substrates such as h = 3 or 4 mm, negligible significant absorption performance is observed across the entire P-band due to potential resonance peaks occurring below 0.3 GHz with increasing thickness of the magnetic substrate. Additionally, Figure 5b presents a schematic diagram illustrating CST simulation for our metamaterial structure, where *Z_max_* represents the surface-emitting electromagnetic waves.

### 3.4. The Exploration of Absorption Mechanism

**Figure 6:**
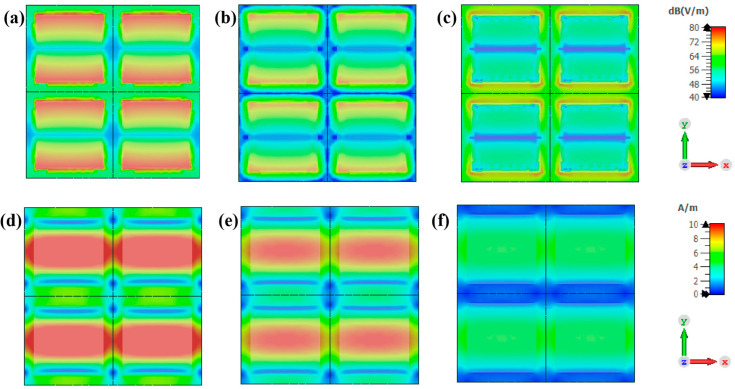
Electric field distribution of metamaterial absorber: (**a**) 0.32 GHz, (**b**) 0.49 GHz, and (**c**) 0.68 GHz. Magnetic field distribution: (**d**) 0.32 GHz, (**e**) 0.49 GHz, and (**f**) 0.68 GHz.

**Figure 7:**
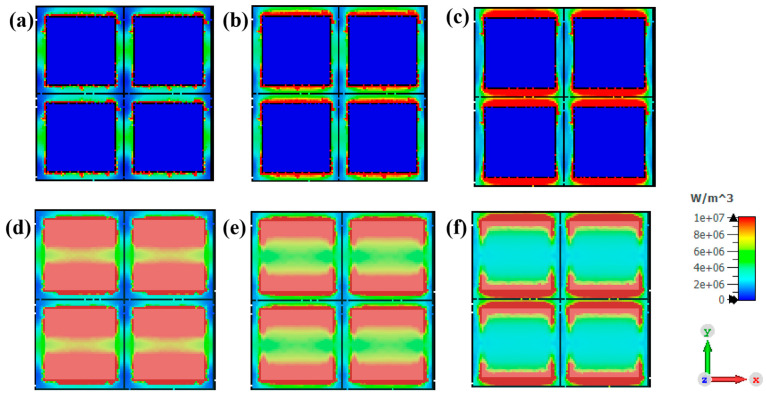
Energy loss density distribution in surface of metamaterials: (**a**) 0.32 GHz, (**b**) 0.49 GHz, and (**c**) 0.68 GHz. Energy loss density distribution between top metal pattern and intermediate basal layer: (**d**) 0.32 GHz, (**e**) 0.49 GHz, and (**f**) 0.68 GHz.

To investigate the absorption mechanism of the designed metamaterial absorber, we monitored the spatial distribution of the electric field, magnetic field (Figure 6), and energy loss density (Figure 7) on the surface of the metamaterial at P-band absorption peak frequencies of 0.32 GHz, 0.49 GHz, and 0.68 GHz. The color scale on the right side of Figure 6 represents a cloud map ranging from blue to red, indicating increasing field strength. At a frequency of 0.32 GHz, the electric field is primarily distributed in both upper and lower parts of the metamaterial pattern layer (Figure 6a), while the magnetic field is predominantly concentrated in its middle section (Figure 6e). This complementary distribution between magnetic and electric fields serves as a prime example of λ/4 characteristic resonance properties [31]. The concept of λ/4 resonance can be understood by analyzing the overlapping of transmitted waves and multiple reflected waves, resulting in the formation of a standing wave within a material. Assuming that along with incident direction, superimposed waves’ electric and magnetic fields are $E_{1y(z,t)\;=\;}E_{0}\cos(kt-wt)$ and $B_{1x(z,t)\;=\;}B_{0}\cos(kt-wt)$, while the electric and magnetic fields of superimposed waves in the opposite direction are altered as $E_{2y(z,t)\;=\;}-E_{0}\cos(kt+wt)$ and $B_{2x(z,t)\;=\;}B_{0}\cos(kt+wt)$. Then, the mathematical formulation representing the standing wave field is provided: $E_{y(z,t)\;=\;}2E_{0}\sin(kt)\sin(wt)$ and $B_{x(z,t)\;=\;}2B_{0}\cos(kt)\cos(wt)$. The expressions for the electric and magnetic fields of standing waves indicate a phase angle of 90 degrees between them, resulting in spatial separation. This characteristic can be utilized to detect the presence of resonance at λ/4. At a frequency of 0.49 GHz, the distribution of electric and magnetic fields remains complementary and consistent with λ/4 resonant behavior. At 0.68 GHz, the electric field is distributed among structural units, demonstrating strong coupling and pronounced diffraction effects within the pattern layer.

The distribution of surface energy loss density in metamaterials (upper row) and the energy loss density between the top metal and intermediate basal layer (lower row) is illustrated in Figure 7. The energy loss density distribution at 0.32 GHz exhibits a similar pattern to that of the electric field distribution map, primarily concentrated in the upper and lower regions of the pattern layer as shown in Figure 7d. Therefore, the energy dissipation at this frequency mainly comes from electrical loss. The energy loss density distribution at 0.49 GHz also shows a close resemblance to the electric field distribution, indicating a strong correlation between them. Additionally, an evident increase in energy loss is observed at the periphery of the metamaterial pattern layer, particularly in the red area depicted in Figure 7b, along with amplified losses between adjacent units. Therefore, it can be concluded that the predominant source of energy dissipation at this frequency primarily stems from electrical losses, augmented by the coupling effect between neighboring structural units induced by strong electric and weak magnetic fields, as well as edge scattering effects. At 0.68 GHz, the energy loss density distribution exhibits a prominent red hue within the interstitial regions between pattern layers. The dissipation of energy at this specific frequency primarily arises from the coupling effect between adjacent structural units and edge scattering effects. Overall, the appearance of characteristic absorption peaks can be attributed to three main factors: λ/4 resonance, coupling effect between adjacent structural units, and scattering effects. By connecting multiple frequency points, the design scheme enables the structure to achieve broadband absorption in the P-band.

### 3.5. Experimental Testing for Absorption Performance in P-Band Testing

**Figure 8:**
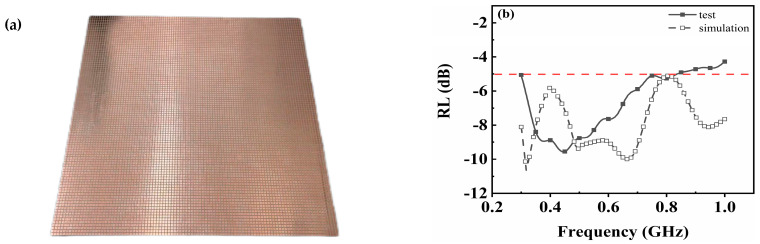
(**a**) Testing sample with a dimension of 1 m × 1 m and pattern units with dimensions of a = 4 mm, b = 1.5 mm, and h = 2 mm. (**b**) Simulated and experimental RL values in the P-band.

Finally, a 1 m × 1 m sample (Figure 8a) was fabricated for P-band absorption performance testing, with dimensions of a = 4 mm, b = 1.5 mm, and h = 2 mm. A comparison was made between the simulation results and the measured values shown in Figure 8b, where the solid line represents the simulated values and the dashed line represents the measured values. In experimental testing, the absorber exhibits absorption resonance peaks at 0.37 GHz and 0.45 GHz, which align closely with the calculated absorption peaks at 0.32 GHz and 0.49 GHz. However, simulation results indicate additional absorption resonance peaks at higher frequencies of 0.68 GHz and 0.95 GHz, resulting in RL values below −5 dB across the entire P-band range. Nevertheless, these last two absorption resonance peaks are absent in actual testing data, leading to a deviation from the expected behavior beyond a frequency of 0.45 GHz, where the reflection loss curve shifts upwards from its minimum point. Moreover, for frequencies exceeding 0.83 GHz, the reflection loss value surpasses the −5 dB threshold, indicating an absence of further absorption performance. The disparity between calculation and experimentation can be ascribed to errors in sample preparation, limitations imposed by experimental conditions, and experimental inaccuracies. The RL value of 1 m specimens is below −5 dB within the frequency range of 0.3–0.83 GHz, exhibiting an effective absorption bandwidth of 0.53 GHz and a maximum reflection loss value of −9.54 dB. The overall thickness of the 1 m specimen measures approximately 2.035 mm, corresponding to roughly 1/293 of the working wavelengths employed. This absorber possesses ultra-thin broadband characteristics.

## 4. Conclusions

After optimizing the four electromagnetic parameters of magnetic materials using GA, the objective function values in CIP magnetic materials can be reduced to below 0.5. When converted to reflectivity, the disparity between the calculated and experimentally measured maximum in reflectivity of a single material is reduced to 0.75 dB.

The combination of the metal capacitive pattern layer and bottom magnetic basal layer in metamaterials facilitates the attainment of low-frequency high-performance absorption. By synergistically adjusting the structural parameters of metamaterials, high-performance absorption performance can be achieved across a wide frequency range in the P-band.

By analyzing the distribution of the electric field, magnetic field, and energy loss density of the absorber at the frequency point of the resonant peak, it can be inferred that the absorption mechanism is attributed to resonance, coupling effect between adjacent structural units, and scattering effects. The presence of multiple connected resonant peaks facilitates broadband absorption of the absorber.

The absorption sample exhibits RL below −5 dB within the 0.3–0.85 GHz frequency range in P-band with a maximum reflection loss value reaching −9.54 dB at 0.45 GHz. Remarkably thin with a thickness of only 1/293 of its operating wavelength, this absorber possesses attributes such as low-frequency operation, broad bandwidth coverage, and ultra-thin structure, thus holding significant potential for military applications.

## Data Availability

Data is contained within the article.

## Supplementary Materials

The following supporting information can be downloaded at: https://www.mdpi.com/article/10.3390/ma17051157/s1, Figure S1: Transmission characteristics of electromagnetic waves in multi-layer materials.
