# Supplementary material for: Ultra-Thin and Broadband P-Band Metamaterial Absorber Based on Carbonyl Iron Powder Composites
Source: Materials (Basel). 2024 Mar 1;17(5):1157. doi: 10.3390/ma17051157 (PMC10934820; doi:10.3390/ma17051157)
Supplement: Supplementary file 1 [file materials-17-01157-s001.zip › materials-2838865-supplementary.pdf]

1. Theoretical model for the calculation of reflectivity in multi-absorbing materials

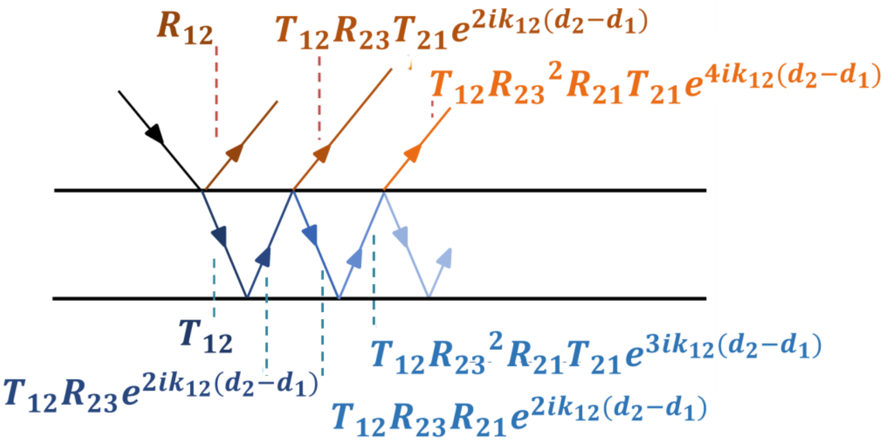

Fig. S1 Transmission characteristics of electromagnetic waves in multi-layer materials

The resonant absorption effect plays a dominant role in multi-layer absorbing materials, and relevant theory is widely applied in the design of absorbing layers. The absorption performance of materials is characterized by their reflection coefficient Γ and reflectivity R, which can be calculated based on transmission line theory.

$$R=20\lg|\Gamma|$$

$$\Gamma=\frac{Z_{\mathrm{in}}(n)-Z_0}{Z_{\mathrm{in}}(n)+Z_0}$$

$$Z_{\mathrm{in}}(n)=\eta_{\mathrm{n}}[\frac{Z_{\mathrm{in}}(n-1)+\eta_{\mathrm{n}}\tanh(\gamma_{\mathrm{n}}\mathrm{d}_{\mathrm{n}})}{\eta_{\mathrm{n}}+Z_{\mathrm{in}}(n-1)\tanh(\gamma_{\mathrm{n}}\mathrm{d}_{\mathrm{n}})}]$$

$$\tanh(x)=\frac{\sinh(x)}{\cos(x)}=\frac{\frac{e^x-e^{-x}}{2}}{\frac{e^x+e^{-x}}{2}}$$

$$\eta_{\mathrm{n}}=\eta_0\sqrt{\frac{\mu_{\mathrm{rn}}}{\varepsilon_{\mathrm{rn}}}}$$

$$\gamma_n=i\frac{2\pi f}{C}\sqrt{\mu_{\mathrm{rn}}\varepsilon_{\mathrm{rn}}}$$

Among them $Z_{\mathrm{in}}(n)$ represents the impedance at the n-th layer interface, η_{n} is the characteristic impedance of the n-th layer material, Z_0 is the air impedance Z_0 =377, γ_{n} is

the propagation constant of the *n*-th layer, d_n is the thickness of the *n*-th layer. *c* is the propagation speed of electromagnetic waves in vacuum, c=3×108 m/s and *f* represents the frequency of electromagnetic wave. Additionally, μ_{rn} and ε_{rn} denote magnetic permeability and dielectric constant of *n*-th layer material respectively. Figure S1 illustrates transmission characteristics of electromagnetic waves in multi-layer materials. For multi-layer structures with metal plates as substrates, inputting impedance between first absorbing material layer and metal plate Zin(0)=0, surface reflectivity can be obtained by using aforementioned formula.
